# Type VII secretion system promotes *Streptococcus agalactiae* virulence through magnesium acquisition and capsule maintenance

**DOI:** 10.1186/s13567-026-01816-9

**Published:** 2026-08-27

**Authors:** Fengyang Li, Chen Xu, Weiyi Ma, Yong Zhou, Yang Zhou, Yong-An Zhang, Hui Zeng

**Affiliations:** 1 Hubei Hongshan Laboratory; Hainan Research Institute; National Key Laboratory of Agricultural Microbiology; Engineering Research Center of Green Development for Conventional Aquatic Biological Industry in the Yangtze River Economic Belt, Ministry of Education; College of Fisheries, Huazhong Agricultural University, Wuhan, 430070 China; 2 Hubei Jiangxia Laboratory, Wuhan, 430200 China; 3 Yangtze River Fisheries Research Institute, Chinese Academy of Fishery Sciences, Wuhan, 430223 Hubei China

**Keywords:** Magnesium, capsule, type VII secretion system (T7SS), *Streptococcus agalactiae*, virulence

## Abstract

*Streptococcus agalactiae*, also known as group B *Streptococcus* (GBS), is a major pathogen causing substantial economic losses in global tilapia aquaculture. The type VII secretion system (T7SS), present in Actinobacteria and Firmicutes, secretes effector proteins implicated in bacterial virulence, yet its functional mechanisms remain poorly defined. Here, we constructed an *essC* deletion mutant (∆*essC*) in *S. agalactiae* strain HN016 to investigate the role of T7SS in virulence. The ∆*essC* exhibited impaired growth and declined intracellular magnesium ion concentration in THB or magnesium-limited chemically defined medium (1 mM Mg^2+^-CDM); these defects were complemented by addition of wild-type culture supernatant. Transcriptomic and quantitative reverse transcription polymerase chain reaction (qRT–PCR) analyses revealed altered sugar metabolism and significant downregulation of capsule biosynthesis genes (*cpsA*, *cpsB*, and *cpsD*) in ∆*essC*. Consistent with this, the mutant produced markedly less capsular polysaccharide and displayed impaired capsule integrity. Furthermore, cellular assays confirmed that compared with HN016, the ∆*essC* mutant exhibited significantly reduced adhesion capacity, immune evasion ability, and cytotoxicity. More importantly, the ∆*essC* mutant showed attenuated virulence in vivo, with reduced bacterial loads in host tissues and diminished ability to cross the blood–brain barrier (BBB). Our findings provide the first evidence that the T7SS influences magnesium homeostasis and is essential for maintaining capsule integrity, both of which contribute critically to pathogenicity. This study identifies T7SS as a potential target for novel therapeutic strategies against streptococcal disease in aquaculture.

**Supplementary Information:**

The online version contains Supplementary Material available at 10.1186/s13567-026-01816-9.

## Introduction

*Streptococcus agalactiae* is a major Gram-positive pathogen responsible for severe streptococcal disease in both freshwater and marine aquaculture, resulting in substantial economic losses due to high mortality rates [1]. Despite its impact, effective therapeutic strategies against *S. agalactiae* remain limited, underscoring the need to elucidate the molecular mechanisms underlying its virulence.

**Figure 1:**
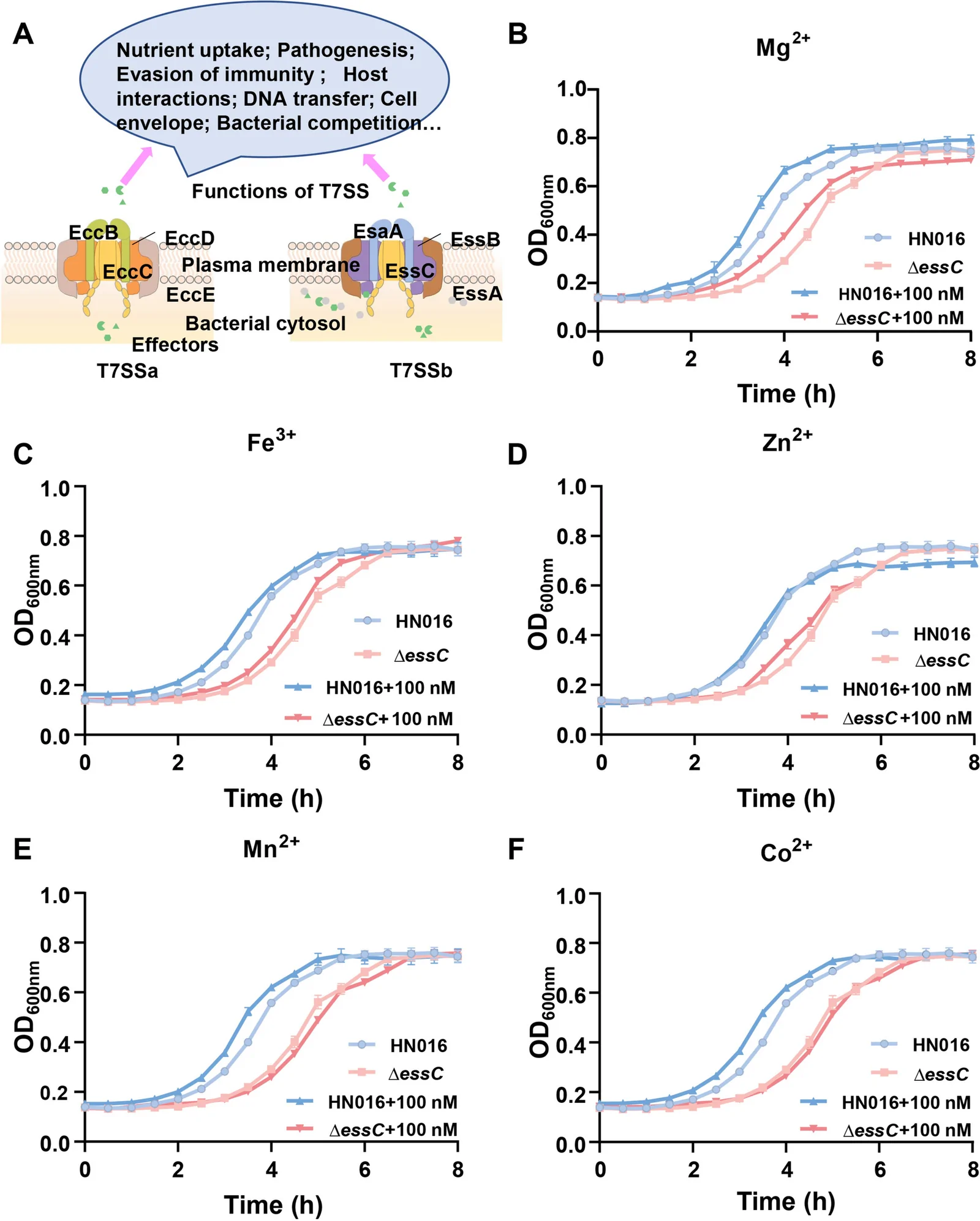
**The ΔessC mutant exhibits a growth defect in magnesium uptake.** **A** Schematic representation of type VII secretion system organization in Actinobacteria (T7SSa) and Firmicutes (T7SSb). Growth curves of HN016 and Δ*essC* mutant were monitored in THB supplemented with 100 nM MgSO_4_ (**B**), Fe_2_(SO_4_)_3_ (**C**), ZnCl_2_ (**D**), MnSO_4_ (**E**), and CoCl_2_ (**F**). Optical density at 600 nm (OD₆₀₀) was measured over time. Data represent mean ± standard error of the mean from at least three independent biological replicates.

Bacterial secretion systems are key virulence determinants that enable pathogens to interact with their environmental, acquire nutrients, exchange genetic material, and promote virulence during infection [2]. To date, 11 distinct types of secretion systems (T1SS to T11SS) have been identified in bacteria [3–6]. The type VII secretion system (T7SS) was first identified in the Gram-positive bacterium *Mycobacterium bovis* BCG [7]. T7SS was divided into T7SSa in Actinobacteria and T7SSb in Firmicutes (Figure 1A) [8]. The T7SSa in Actinobacteria, best exemplified by the ESX-1 to ESX-5 systems in *Mycobacterium tuberculosis* (Mtb), has been well characterized. These systems play established roles in nutrient acquisition and host–pathogen interaction, primarily through the secretion of diverse effector proteins [9]. For example, the ESX-1 system secretes cytolytic effectors that are crucial for macrophage escape and virulence [10], while the ESX-3 system is dedicated to iron acquisition, a vital process for intracellular survival [11]. Furthermore, the ESX-5 system contributes to capsule integrity and pathogenicity [12], with the mycobacterial capsular layer known to incorporate proteins secreted via the ESX-1 pathway [13]. Beyond these, Mtb secretes additional T7SS substrates, including PE12, PE18, PE19, EsxG, EsxS, EspC, and CFP10, which directly target host macrophages to modulate immune responses [14]. In stark contrast to the well-defined functions of T7SSa, the role of its counterpart T7SSb in Firmicutes remains largely unexplored. Limited studies suggest its involvement in virulence, as evidenced by *S. agalactiae* utilizing T7SS, which belong to T7SSb, to secrete EsxA proteins that contribute to cytotoxicity [2]. In *Staphylococcus aureus*, the T7SSb substrate TspA acts as a membrane-depolarizing toxin mediating intraspecies competition [15]; EsxB binds to the STING protein and thereby inhibits the inflammatory defense mechanism [16]. These preliminary findings, however, represent isolated examples rather than a systematic understanding. Therefore, the specific mechanisms by which T7SSb modulates virulence, particularly regarding nutrient acquisition and virulence regulation, remain to be fully elucidated.

Metal ions are crucial cofactors for numerous cellular processes and bacterial pathogenesis [17]. In T7SSa, the ESX-3 contributes to iron acquisition via secreted substrates PE5–PPE4. By contrast, whether T7SSb fulfills an analogous role in metal homeostasis remains unknown. The structural conservation between these systems, particularly in their core membrane-embedded ATPases (EccC in T7SSa and EssC in T7SSb), suggests a potential shared function in substrate secretion and metal acquisition [18]. To test the hypothesis that T7SS contributes to metal ion uptake and virulence in *S. agalactiae*, we constructed an *essC*-deletion mutant (∆*essC*). Here, we demonstrate that EssC is required for maintaining magnesium homeostasis and preserving capsular integrity, two processes essential for full virulence. Our findings establish T7SS as a key mediator of metal acquisition in *S. agalactiae* and reveal new targets for further development of antibacterial drugs.

## Materials and methods

### Bacterial strains, cell lines, and experimental animals

The *S. agalactiae* strain HN016 was isolated in 2010 from a moribund cultured tilapia in China’s Hainan Province [19]. *S. agalactiae* strains were cultured statically at 37 °C in Todd–Hewitt broth (THB) (Hopebio); *Escherichia coli* strains were grown in Luria Bertani (LB) broth (Hopebio) at 37 °C with rotation at 220 rpm. Spectinomycin (Spc; Biofroxx) at 100 µg/mL contained the media to select for plasmids pSET4s or pSET2, whereas no antibiotics were present during phenotypic assays. RAW264.7 murine macrophages were cultured in Dulbecco’s modified Eagle medium (DMEM; Gibco) with 10% fetal bovine serum (FBS; Yeasen) at 37 °C under 5% CO₂, while human brain microvascular endothelial cells (hBMECs) were maintained under the same temperature in Roswell Park Memorial Institute (RPMI) 1640 medium (Gibco) supplemented with 10% FBS.

Healthy Nile tilapia (*Oreochromis nilotica*, averaging 10 ± 1 g in body weight) were purchased from Yue Qiangfeng Hatchery (Guangdong, Guangzhou, China). Prior to experiments, fish were acclimated for at least 2 weeks in 300-L recirculating tanks with filtered, oxygenated water maintained at 28 ± 1 °C. All fish were fed daily at a rate 1.5% body weight with commercial pellet feed (Haida, China) and were examined externally to confirm the absence of clinical signs of disease.

### Standardized molecular biology techniques

All polymerase chain reactions (PCR) were performed using Prime STAR Max DNA Polymerase (Takara, cat. no. R045A). Purification of PCR and restriction digest products was performed using the HiPure Gel Pure DNA Mini Kit (Magen, cat. no. D2111-02). Plasmids were extracted using Hipure plasmid/BAC EF mini kits (Magen, cat. no. P1154-02). Restriction digestions were carried out at 37 °C for a minimum of 30 min using enzymes from Takara. The fusion fragment and vector pSET4s were digested with Sac *I* and Sal *I*, whereas plasmid pSET2 was digested with Pst *I* and EcoR *I* for pSET2-*essC* or Sph *I* and EcoR *I* for pSET2-uridine phosphorylase-EGFP. Ligations used 2× MultiF Seamless Assembly Mix (Abclonal, cat. no. RK21020) at 50 °C for 15 min. All constructs were verified by Sanger sequencing, with the primers used detailed in Additional file 1.

### Mutant strain construction

The *essC* deletion mutant Δ*essC* was generated in *S. agalactiae* HN016 by allelic exchange using the temperature-sensitive plasmid pSET4s, which carried a spectinomycin resistance gene. The ~1 kb homology arms upstream and downstream of the *essC* locus were fused by overlapping PCR and subsequently cloned into pSET4s, generating the *essC* deletion plasmid pSET4s-*essC*. The plasmid was then introduced into HN016 by electroporation. Single-crossover mutants were isolated on Spc-containing THB agar (100 µg/mL) at 28 °C. Putative double-crossover mutants were subsequently screened by replica plating for temperature sensitivity (instability to grow at 37 ℃ due to loss of the temperature-sensitive plasmid pSET4s) and spectinomycin sensitivity [20]. For complementation, the full length *essC* gene was amplified from HN016 genomic DNA and cloned into the *E. coli* and *Streptococcus* shuttle vector pSET2. The plasmid pSET2-*essC* was electroporated into the Δ*essC* mutant to generate the complement strain CΔ*essC*.

### Growth curve

Bacterial cultures with an OD_600_ = 0.65 were washed three times with phosphate-buffered saline (PBS) and diluted 1:500 in either THB or THB supplied with 100 nM of filter-sterilized (0.22 µm) metal salts: Fe₂(SO_4_)_3_, ZnCl_2_, MnSO_4_, CoCl₂, or MgSO_4_. For growth in defined medium, a chemically defined medium (CDM) was prepared according to established methods [21], with MgSO_4_ omitted from the base formulation for Mg^2+^-limiting conditions and added back at specified concentrations (1 mM) prior to sterile filtration. Growth was monitored in a 96-well microplate containing 200 μL of culture pre-well, with three technical replicates per condition. The microplate was incubated at 37 ℃ in a plate reader (BioTek Synergy HTX), and the OD_600_ were recorded hourly. Data from replicates were averaged and plotted as the OD_600_ versus time.

### Intracellular magnesium quantification

Intracellular magnesium levels were quantified using the fluorescent magnesium indicator Mag-Fluo-4AM (Maokang, cat. no. MX4544, China). Briefly, mid-log-phase bacteria were collected by centrifugation (3000*g*, 10 min) and washed three times with PBS. The pellets were resuspended in PBS containing 1 μM Mag-Fluo-4AM and 1.25 μL 20% Pluronic F-127 (MaoKang, cat. no. MS4302) to facilitate dye loading. After dark incubation at 37 °C for 30 min, cells were washed thrice with PBS to remove extracellular dye. Using a microplate reader (BioTek Epoch 2, USA), fluorescence was measured. To correlate fluorescence with bacterial number, the OD₆₀₀ of each sample was measured in parallel. Intracellular magnesium levels were expressed as mean fluorescence intensity per unit OD_600_. Each experiment included three technical replicates and was performed independently three times.

### RNA sequencing and analysis

Following overnight culture in THB at 37 °C, *S. agalactiae* HN016 and the Δ*essC* mutant were diluted 1:100 in 10 mL of fresh THB and grown at 37 °C to an OD_600_ of 0.6. Cells from three independent biological replicates per strain were harvested by centrifugation. Total RNA was extracted with TRIzol^®^ Reagent (Invitrogen) per the manufacturer’s instructions, followed by library preparation and 150-base pair (bp) paired-end sequencing on an Illumina platform at the Microbial Genome Sequencing Center. Reads were mapped to the HN016 genome (NZ_CP011325.1) through Rockhopper. Using the DESeq2 package, differential gene expression was analyzed, with genes considered significant at an adjusted *p* < 0.05 and |log₂ fold change| > 1. For validation, quantitative reverse transcription polymerase chain reaction *(qRT–PCR)* was performed on selected genes using the primers listed in Additional file 1. Primers for *gyrA* (internal control), *cpsA*, *cpsB*, *cpsD*, and *cpsE* were all used as previously described [22]. Transcript levels were normalized to *gyrA* expression using the 2^−ΔΔCT^ method.

### Capsule protein extraction and mass spectrometry (MS) analysis

Capsule proteins were extracted following the protocol [13] with slight adaptations. Following growth to OD_600_ = 0.65 (as above), bacteria were pelleted, washed three times with PBS, and resuspended in PBS with 0.5% Genapol X-080 (Beyotime, ST1344) for a 1-h incubation at 28 °C with gentle agitation. The collected supernatant (capsular fraction) was treated with 10% (vol/vol) trichloroacetic acid (TCA) and incubated on ice for 30 min. Following centrifugation (12,000*g*, 20 min) to precipitate proteins, the pellet was washed twice with cold acetone and air-dried in a fume hood. Following separation by sodium dodecyl sulfate–polyacrylamide gel electrophoresis (SDS–PAGE) and Coomassie blue staining, differential protein bands between HN016 and Δ*essC* were excised. These gel pieces were then in-gel digested with trypsin (1:50, 37 °C, overnight) and analyzed by liquid chromatography–tandem mass spectrometry (LC–MS/MS) using a Thermo LTQ FT. MS data were searched against the *S. agalactiae* HN016 protein database using proteome discoverer with tryptic specificity and a false-discovery rate (FDR) of < 1%.

Capsular sialic acid content was quantified from the Genapol X-080-extracted supernatant using a commercial sialic acid assay kit (BaiAoLaiBo, cat. no. SNM163) following the standard protocol.

### Scanning electron microscope (SEM) and super-resolution fluorescence microscopy imaging

The samples for SEM were prepared following an established protocol [23] with minor modifications. Briefly, cultures (HN016 and Δ*essC*) were grown to OD_600_ = 0.65, harvested, PBS-washed, and fixed (2.5% glutaraldehyde, 4 °C, overnight). Following PBS washes, samples were dehydrated in a graded ethanol series, critical-point dried, and sputter-coated with gold. The images were captured using a field-emission scanning electron microscope (FESEM; Techcomp, China).

For super-resolution fluorescence microscopy, the pSET2-uridine phosphorylase-EGFP plasmid was electroporated into HN016 to generate a strain that expresses a functional fusion protein, and then cultured to an OD_600_ = 0.65. Cells were harvested, and the capsule was labeled with anti-serotype Ia CPS antiserum (1:2000; Wuhan Jianhao Biological Technology, cat. no. CBIa11) and a Cy3-conjugated goat anti-rabbit IgG (H + L) secondary antibody (1:100, ABclonal, cat. no. AS007). Cells were immobilized on coverslips coated with poly-l-lysine and fixed with 4% paraformaldehyde. Subsequently, images were acquired on a Leica TCS SP8 STED 3X super-resolution microscope using a 100× oil-immersion objective. EGFP and Cy3 were excited with a 488-nm or 550-nm laser. Image processing and analysis were performed using LAS X (Leica) and ImageJ software.

### Adherence and cell toxicity assays

Tilapia brain cells( TiB ), tilapia head kidneys (HKLs), and RAW264.7 murine macrophages were seeded in 24-well plates (VWR, cat. no. 734–2325) and plated at 1.5 × 10^5^ cells per well and allowed to adhere until confluent monolayers formed. *S. agalactiae* strains with an OD_600_ = 0.65 were washed and resuspended in PBS. Bacterial concentrations were normalized by OD_600_ and confirmed by plating to determine colony-forming units (CFU). In adherence assays, cells were infected at a multiplicity of infection (MOI) of 10 for 30 min (37 °C, 5% CO₂) after a PBS wash. Following infection, monolayers were PBS-washed and lysed in sterile water, and the resulting lysates were serially diluted and plated on THB agar for CFU quantification. For cytotoxicity assays, cells were infected (MOI = 10, 4 h). Supernatants were analyzed for LDH release with a Beyotime kit (cat. no. C0016) according to the manufacturer’s protocol. Cytotoxicity was expressed as a percentage relative to the maximum LDH release obtained from cells lysed with the kit lysis buffer.

To assess contact-independent cytotoxicity, bacteria were separated from host cells using 0.4 µm polycarbonate Transwell inserts (Corning), as previously described [2]. After 4 h of incubation, LDH release in the lower chamber was measured as above. All assays were performed in three independent biological replicates, each in technical triplicates.

### Blood serum and leukocyte of tilapia bactericidal assays

Tilapia were anesthetized with MS-222 (100 mg/L) for blood collection from the caudal vein. Following collection, serum was separated by centrifugation (3500 *g*, 10 min) after overnight clotting at 4 °C. Blood serum killing assay was carried out following an established protocol [20] with minor modifications. Mid-log-phase *S. agalactiae* cells (1 × 10^3^ CFU) were incubated with 50 µL of fresh tilapia serum (or PBS as a control) at 37 °C for 1 h. Serially diluted samples were plated on THB agar and incubated at 37 °C for 12 h prior to colony counting. The survival rate was expressed as (CFU in serum/CFU in PBS) × 100%. For leukocyte killing assays, leukocytes were isolated from tilapia head kidneys (HKLs) following the method [24–27] with slight adaptations. Briefly, head kidney leukocytes (HKLs) were isolated by tissue dissociation, filtration (100 μm), and centrifugation on a 34%/51% Percoll gradient (400 *g*, 30 min, 4 °C). Collected interface cells were washed in DMEM and resuspended in complete DMEM containing 10% FBS. Isolated HKLs were seeded in 24-well plates (2 × 10^5^ cells/well) and allowed to adhere for 4 h at 28 °C. *S. agalactiae* was resuspended in DMEM and added to the leukocytes at an MOI of 10. After 120 min or 180 min of co-incubation at 28 °C, serial dilutions of the supernatant were plated on THB agar to assess bacterial survival. The killing rate was calculated as [1 − (CFU at time *t*/CFU at time 0)] × 100%.

### Tilapia infection and tissue analysis

Tilapia infection was performed as described previously [28]. Briefly, HN016 and Δ*essC* were grown to OD_600_ = 0.65, washed, and resuspended in PBS, and their concentrations were verified by plating serial dilutions on THB. Healthy Nile tilapia (average weight 10 ± 1 g) were casually allocated into three groups (*n* = 30 per group): (1) wild-type (WT) HN016, (2) Δ*essC* mutant, and (3) PBS control. Fish were infected via intraperitoneal injection with 1 × 10^8^ CFU in 100 µL PBS (or PBS alone for controls). Survival was recorded daily for 14 days, with the data plotted as survival curves.

To assess systemic colonization, a separate cohort of fish (*n* = 5 per group) was infected intraperitoneally with 1 × 10^4^ CFU/fish. At 96 h post-infection, brain, liver, spleen, head kidney, trunk kidney, and hindgut were aseptically collected. Tissues were homogenized in PBS (1 mL) using a tissue grinder. Bacterial loads (CFU/g) were quantified by plating serial dilutions of homogenates on THB. Parallel samples were fixed in 4% paraformaldehyde (Servicebio, 24 h, 4 °C), then paraffin-embedded, sectioned (5 µm), and hematoxylin and eosin (H&E)-stained for blinded histopathological examination under a light microscope.

### Evans blue analysis

The permeability of the blood–brain barrier (BBB) was determined by Evans blue (EB) extravasation, as described previously [29]. Tilapia were intraperitoneally infected with HN016 or Δ*essC* (1 × 10^5^ CFU/fish); at 4 h post-infection, 200 µL of 2% EB was administered via caudal vein injection. After 30 min, animals were anesthetized and perfused transcardially with saline for dye removal. The harvested brains were photographed and homogenized in 1 mL of formamide. The homogenates were incubated at 37 °C for 72 h and centrifuged, and the supernatant absorbance was measured at 620 nm. EB concentration was calculated against a standard curve and normalized to brain weight.

To assess bacterial penetration of the endothelial barrier, hBMECs were seeded onto 0.4-µm pore polycarbonate Transwell inserts (Corning) and grown to confluent monolayers. Monolayers were infected apically with HN016 or Δ*essC* at an MOI of 10. After 12 h at 37 °C, the inserts were washed three times with PBS, and 100 µL of 2% EB was added to the apical chamber. Following 30 min of incubation, the medium in the basolateral chamber was collected, and EB was extracted with formamide as described above. Absorbance was measured at 620 nm, and EB translocation was expressed as a percentage of the total dye added.

### Statistical analysis

Results are expressed as mean ± standard error of the mean from a minimum of three biological replicates. All statistical tests, including Student’s *t*-test for pairwise comparisons, one-way analysis of variance (ANOVA) for multi-group comparisons, and the Kaplan–Meier method for survival analysis, were conducted using GraphPad Prism software. Statistical significance, indicated directly in figures, was set at *p < 0.05, **p < 0.01, ***p < 0.001, and ****p < 0.0001.

## Results

### T7SS facilitates the transport of magnesium ions in *S. agalactiae* HN016

The organization and function of the type VII secretion system in Actinobacteria (T7SSa) and Firmicutes (T7SSb) is schematically shown in Figure 1A. Structural modeling indicated that the core ATPase of the T7SS is conserved between *S. agalactiae* and *M. tuberculosis* (Additional file 2A, B). To investigate its function in *S. agalactiae*, we constructed an *essC* deletion mutant (Δ*essC*) and a complement strain (CΔ*essC*), as validated by PCR and sequencing (Additional file 2C–E). Initial phenotypic screening in THB medium revealed no significant difference between WT and CΔ*essC* (Additional file 3), whereas the Δ*essC* mutant exhibited a prolonged lag phase compared with the WT HN016 (Figure 1B). This growth delay was specifically rescued by supplementation with 100 nM Mg^2+^ (Figure 1B) but not by equimolar concentrations of Fe^3+^, Zn^2+^, Mn^2+^, or Co^2+^ (Figure 1C–F), suggesting a selective role for T7SS in magnesium homeostasis.

**Figure 2:**
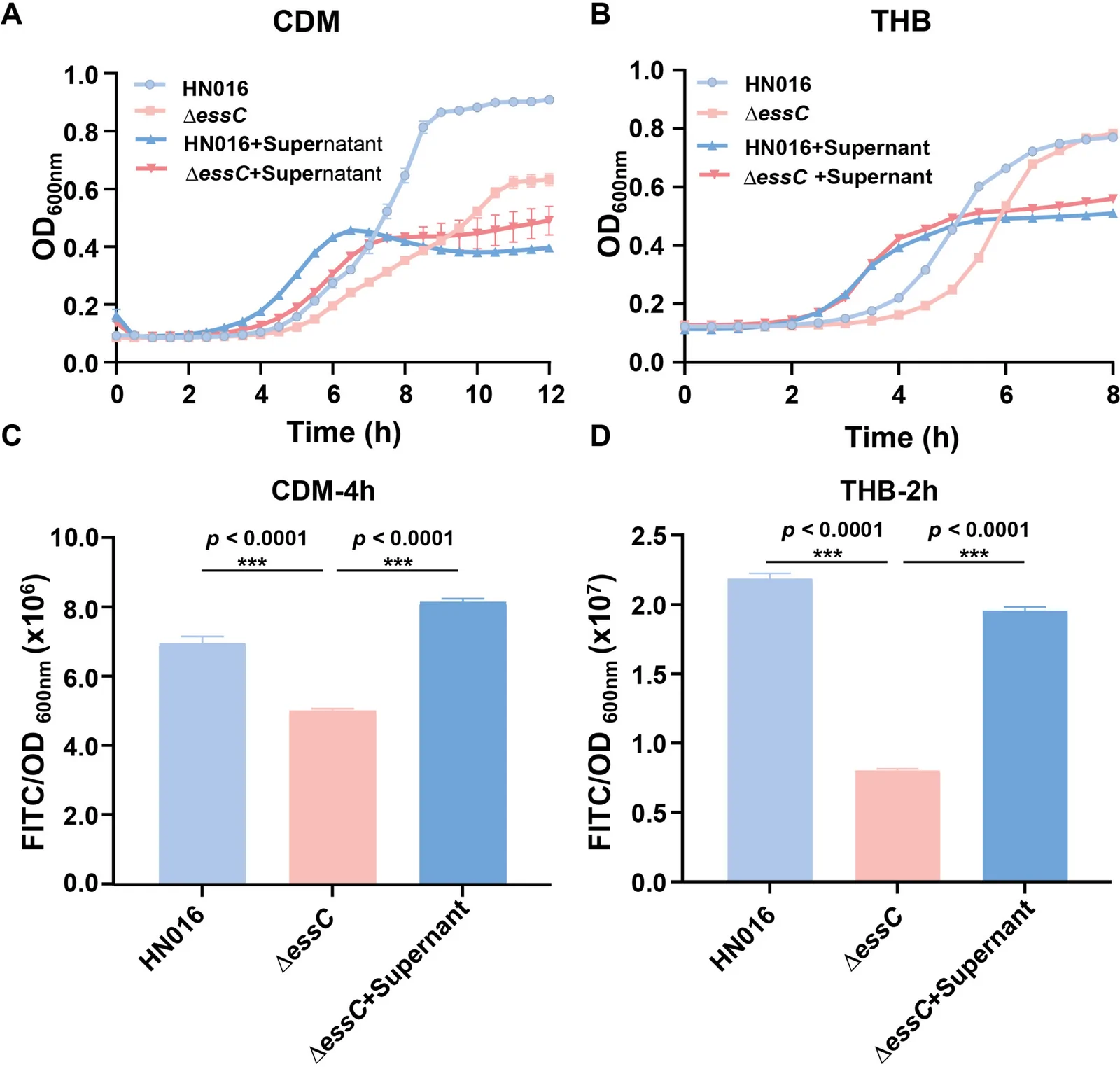
**The T7SS is essential for magnesium acquisition in** ***S. agalactiae***. **A** Growth curves of HN016 and the Δ*essC* mutant in CDM with or without 1 mM MgSO₄, or 50% (vol/vol) filter-sterilized supernatant from HN016 cultures. **B** Growth curves of HN016 and the Δ*essC* mutant in rich medium (THB) with or without 1 mM MgSO₄, or 50% (vol/vol) filter-sterilized supernatant from HN016 cultures. OD₆₀₀ was measured over time. Intracellular magnesium levels measured by Mag-Fluo-4 AM fluorescence in HN016 and the Δ*essC* mutant cultured in CDM for 4 h (**C**) or in THB for 2 h (**D**), with or without supplementation of 50% (vol/vol) HN016 supernatant. Fluorescence intensity was normalized to OD₆₀₀. Data represent mean ± standard error of the mean from three independent experiments. *p < 0.05, **p < 0.01, ***p < 0.001 (Student’s *t*-test).

To further investigate the impact of T7SS on Mg^2+^ homeostasis, the growths of WT and Δ*essC* under defined magnesium-limited conditions were examined. In CDM containing 1 mM Mg^2+^, the Δ*essC* mutant displayed a pronounced lag and a reduced growth rate, resulting in delayed entry into stationary phase and lower final cell density (Figure 2A). Supplementation of the mutant with filter-sterilized supernatant from WT cultures fully restored its growth kinetics in both CDM (Figure 2A) and THB (Figure 2B), indicating that the defect is complemented by a secreted factor. Consistent with the growth phenotype, intracellular magnesium levels were significantly lower in the Δ*essC* mutant after 4 h in CDM (Figure 2C) and after 2 h in THB (Figure 2D). Importantly, addition of WT supernatant restored intracellular magnesium compared with Δ*essC* (Figure 2C, D). Together, these data establish that T7SS in *S. agalactiae* is required for efficient magnesium acquisition, likely through the secretion of an effector that enhances magnesium availability to the cell.

### T7SS disruption remodels glycolytic and capsule biosynthesis pathways

**Figure 3:**
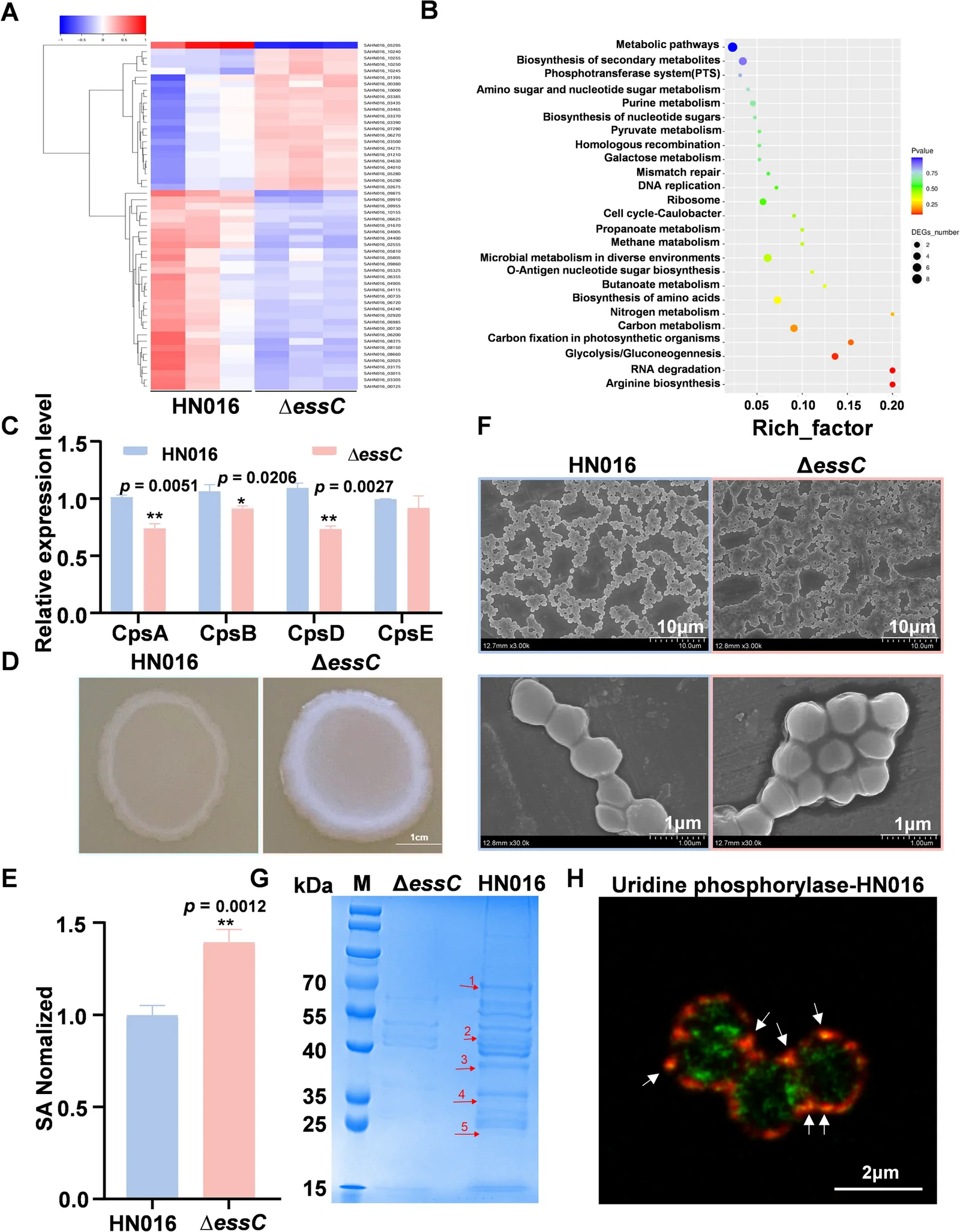
**T7SS is essential for maintaining capsular integrity in** ***S. agalactiae***. **A** DEGs in the Δ*essC* mutant versus HN016. Color intensity reflects log_10_ (FPKM) values, with red and blue indicating higher and lower expression, respectively. **B** KEGG pathway enrichment analysis of DEGs identified in **A**. **C** qRT‑PCR validation of selected capsule biosynthesis genes (*cpsA*, *cpsB*, *cpsD*) in HN016 and the Δ*essC* mutant. Expression levels are normalized to the housekeeping gene *gyrA*. Data are mean ± standard error of the mean of three biological replicates (*p < 0.05, **p < 0.01, Student’s *t*‑test). **D** Colony morphology of HN016 and the Δ*essC* mutant plated at 5 × 10^6^ CFU. A prominent “halo” (indicated by an arrow) is visible around Δ*essC* colonies. **E** Quantification of surface associated sialic acid, a major capsule component, in HN016 and the Δ*essC* mutant (**p < 0.01, Student’s *t*‑test). **F** Scanning electron microscopy (SEM) images of HN016 (left) and the Δ*essC* mutant (right). The mutant exhibits cell clumping and surface irregularities. Scale bars: 10 μm (overview), 1 μm (inset). **G** SDS–PAGE analysis of capsular protein extracts from HN016 and the Δ*essC* mutant. Arrows mark protein bands markedly reduced in the mutant. M: protein molecular weight marker. **H** Subcellular localization of uridine phosphorylase (identified in panel **G**) visualized by super‑resolution fluorescence microscopy. Uridine phosphorylase‑EGFP (green) co‑localizes with the capsule stain (red) in the merged image (orange). Scale bar: 2 μm.

To investigate the molecular basis of the growth defect observed in the Δ*essC*, we performed RNA sequencing (RNA-seq) analysis comparing the transcriptomes of the Δ*essC* mutant and WT HN016 strain. A total of 54 differentially expressed genes (DEGs) were identified, with 22 upregulated genes and 32 downregulated genes in the Δ*essC* strain (Figure 3A). The significant downregulation of the known T7SS effector gene *esxA* (3.5-fold) confirmed the expected impact of the *essC* deletion. Among the downregulated genes, multiple key glycolytic enzymes were underrepresented, including type I glyceraldehyde-3-phosphate dehydrogenase (SAHN016_08660), formate C-acetyltransferase (SAHN016_08375), phosphocarrier protein HPr (SAHN016_04400), and triose-phosphate isomerase (SAHN016_04115). Strikingly, several genes critical for capsule biogenesis and maintenance of cell wall integrity, such as *cpsA* (SAHN016_06010), *cpsB* (SAHN016_06005), *cpsD* (SAHN016_05995), *cpsE* (SAHN016_05990), and LysM peptidoglycan-binding domain-containing protein (SAHN016_00320), were also markedly downregulated. Kyoto Encyclopedia of Genes and Genomes (KEGG) enrichment analysis further highlighted that the DEGs were related to sugar metabolism, including the phosphotransferase system (PTS), amino sugar and nucleotide sugar metabolism, purine metabolism, biosynthesis of nucleotide sugars, pyruvate metabolism, and galactose metabolism (Figure 3B). *qRT-PCR* validation of selected genes confirmed the RNA-seq findings. Seven genes (*cpsA*, *cpsB*, *cpsD*, *spx*, *SAHN016*_*03175*, *gap*, and *raiA*) were significantly downregulated in the Δ*essC*, whereas *cpsC* showed no significant changes, demonstrating high consistency between the two platforms (Figure 3C and Additional file 4). Collectively, these transcriptomic data indicate that EssC is required for the normal expression of glycolytic and capsular polysaccharide synthesis genes, linking T7SS function to central carbon metabolism and surface structure integrity.

### T7SS promotes capsular integrity

Given the transcriptional downregulation of capsule biosynthesis genes and altered glycolytic pathways in the Δ*essC* mutant, we hypothesized that T7SS disruption compromises capsule biogenesis. On the solid THB medium, the Δ*essC* colonies were surrounded by a conspicuous white ring (Figure 3D), a phenotype absent in the WT HN016, suggesting potential capsule instability or shedding. Consist with this, surface associated sialic acid, one of the components of capsular polysaccharides [30], was significantly elevated in the mutant (1.41-fold increase, *p* = 0.0012, Figure 3E), further indicating altered capsular architecture. SEM revealed that Δ*essC* cells exhibited pronounced clumping (Figure 3F), a morphology that is often associated with compromised capsule integrity.

Given the observed reduction in capsular biosynthesis proteins in Δ*essC* mutant, and inspired by the precedent that the *M. tuberculosis* capsule is enriched with proteins secreted via the ESX-1 (T7SSa) system[13], we next asked whether T7SS in *S. agalactiae* similarly contributes the essential proteins to its capsule. Comparative SDS–PAGE analysis of extracted capsular fractions identified six distinct protein bands that were markedly reduced in the Δ*essC* mutant (bands 1–6, Figure 3G). Mass spectrometry of these bands identified several candidates, such as uridine phosphorylase, adenylate kinase, DUF1002 domain-containing protein, fructose-6-phosphate aldolase, and phosphoserine phosphatase SerB (Additional file 5–9). To validate the MS findings, we expressed a functional EGFP-tagged uridine phosphorylase protein in WT HN016 and, as expected, observed its presence both in the cytoplasm and the capsular compartment using super-resolution fluorescence (Figure 3H). Collectively, these data demonstrate that T7SS is required for maintaining capsular integrity in *S. agalactiae* by facilitating the proper secretion or retention of proteins essential for capsular integrity.

### T7SS is essential for *S. agalactiae* adherence, serum resistance, and cytotoxicity

**Figure 4:**
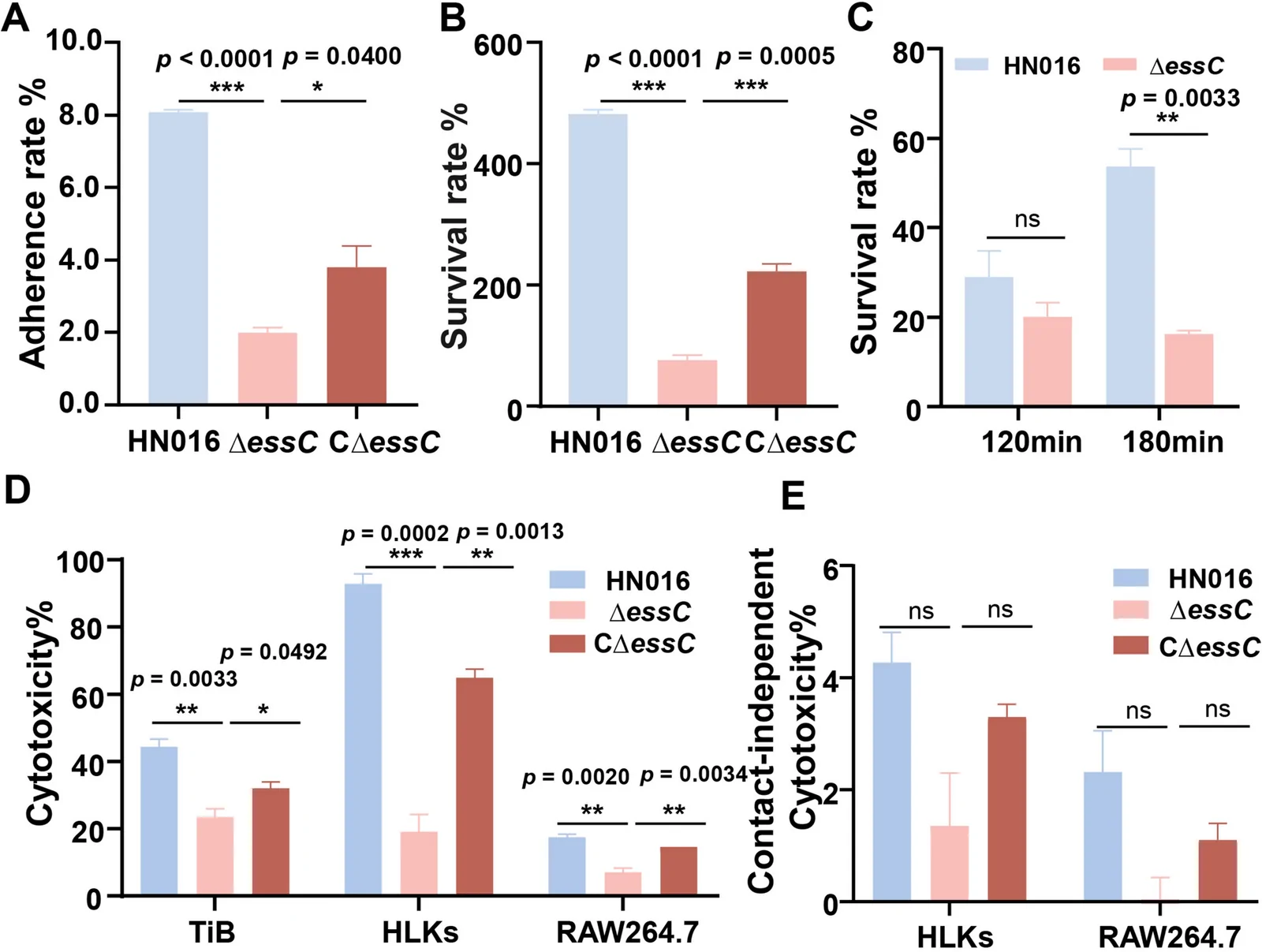
**T7SS is required for host cell adhesion, serum resistance, and contact-dependent cytotoxicity.** **A** Adhesion of HN016, Δ*essC* mutant, and CΔ*essC* to TiB cells. **B** Serum resistance assay. Survival of HN016 and Δ*essC* after 1 h of incubation in serum from healthy tilapia. **C** Bacterial survival following co‑culture with tilapia head kidney leukocytes (HKLs) for 120 and 180 min. **D** Cytotoxicity toward TiB, HKLs, and RAW264.7 cells measured by LDH release after 4 h of infection at an MOI of 10. Percent cytotoxicity was normalized to fully lysed (100%) controls. **E** Cytotoxicity toward HKLs and RAW264.7 cells in a Transwell system in which bacteria were physically separated from host cells by a 0.4-μm porous membrane. Cytotoxicity was measured after 4 h. Data are presented as mean ± standard error of the mean of three independent experiments. Statistical significance was assessed by repeated‑measures one‑way ANOVA with Tukey’s multiple‑comparison test (*p < 0.05, **p < 0.01, ***p < 0.001). Data represent mean ± standard error of the mean of three independent experiments.

Given the central role of capsule in virulence, we next assessed how its structural compromise in the Δ*essC* mutant affects key host–pathogen interactions. Adherence assays using TiB cells demonstrated that the Δ*essC* mutant exhibited significantly reduced adherence compared with both WT HN016 and CΔ*essC* (Figure 4A). To evaluate serum resistance, we exposed bacteria to serum form healthy tilapia. While the WT HN016 strain exhibited robust proliferation, achieving approximately 530% survival relative to the initial inoculum, the Δ*essC* mutant showed markedly impaired growth, with survival limited to only 80% (Figure 4B). The CΔ*essC* strain displayed an intermediate phenotype, recovering to approximately 200% survival (Figure 4B). Similarly, the Δ*essC* strain was more efficiently cleared by tilapia HKLs, exhibiting a survival rate of only 16.25% compared with 53.68% for HN016 after 180 min of co-incubation (Figure 4C).

We further evaluated bacterial cytotoxicity toward TiB, HKLs, and RAW264.7 cells. Infection with WT HN016 and CΔ*essC* caused approximately 42% and 31% cytotoxicity to TiB cells, respectively, whereas the Δ*essC* mutant induced significantly less cell death (~20%) (Figure 4D). The Δ*essC* mutant consistently displayed reduced cytotoxicity in two other cell types, causing ∼6% (RAW264.7 cells) and ∼20% (HKLs) LDH release, compared with ∼17% and ∼90% for HN016, respectively (Figure 4D). Furthermore, a Transwell assay that physically separated bacteria from host cells resulted in minimal LDH release across all strains (less than 2.5% for RAW264.7 and less than 5% for HKLs), suggesting that T7SS-mediated cytotoxicity in HN016 is predominantly contact-dependent (Figure 4E). Collectively, these findings demonstrate that T7SS is critical for evading humoral immunity and cytotoxicity in *S. agalactiae*.

### T7SS is essential for *S. agalactiae* virulence in tilapia

**Figure 5:**
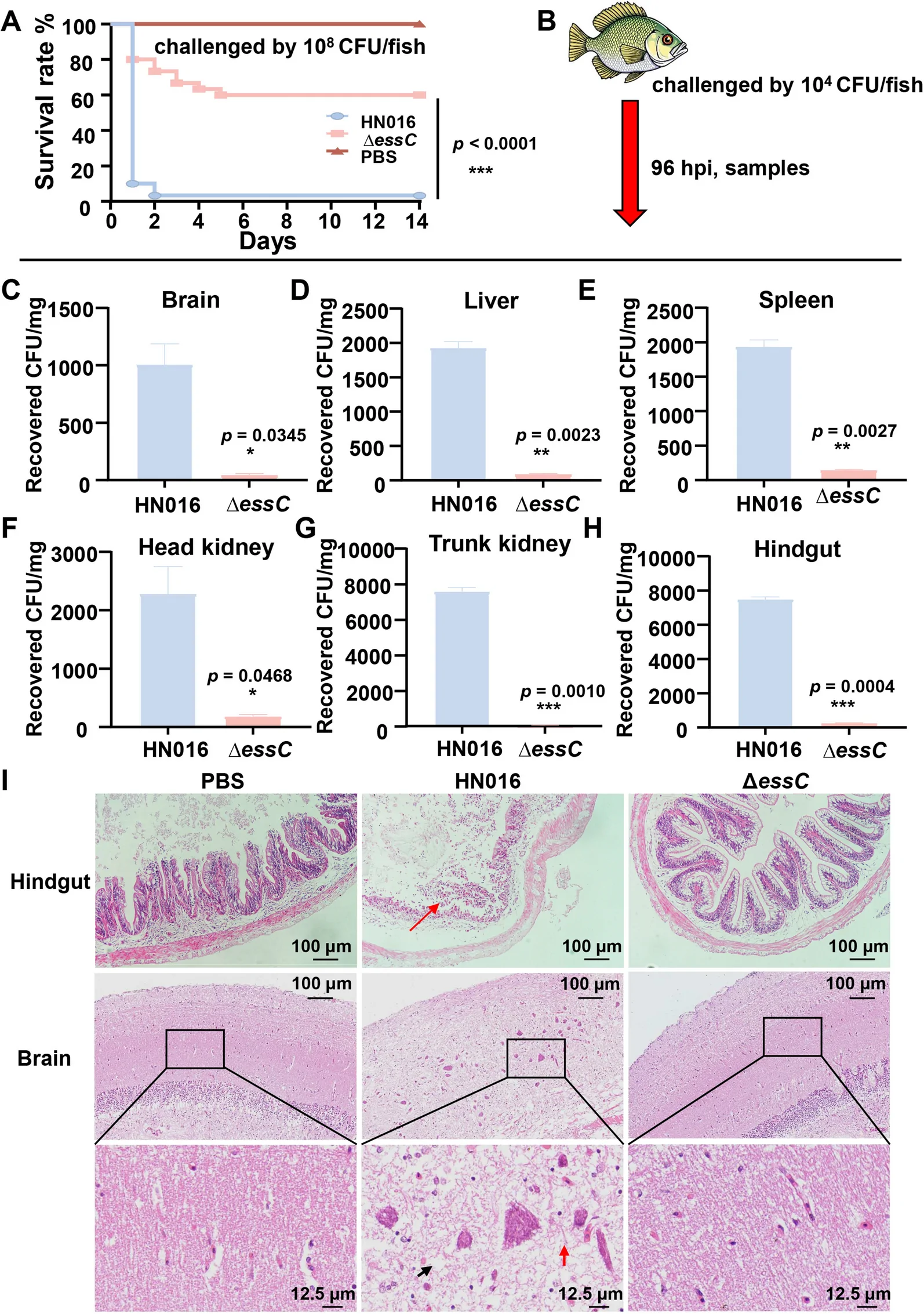
**T7SS is essential for systemic virulence and tissue colonization in a tilapia infection model.** **A** Kaplan–Meier survival analysis of tilapia (10 ± 1 g) following intraperitoneal injection with 1 × 10⁸ CFU/fish of HN016, Δ*essC* mutant, or PBS (*n* = 30 per group). **B** Schematic of the experimental challenge design. **C–H** Bacterial loads (CFU/organ) in the (**C**) brain, (**D**) liver, (**E**) spleen, (**F**) head kidney, (**G**) trunk kidney, and (**H**) hindgut at 96 h post‑infection (inoculum: 1 × 10^4^ CFU/fish; *n* = 3 fish per group). Data are presented as mean ± standard error of the mean. Statistical significance was determined by Student’s *t*‑test (*p < 0.05, **p < 0.01, ***p < 0.001). **I** Histopathological analysis (H&E staining) of the hindgut (upper panels) and brain (lower panels) from tilapia infected for 96 h with PBS (control), HN016, or the Δ*essC* mutant. Arrows highlight representative lesions, including the red arrow in HN016 representing the loose and disorganized cerebral parenchyma and the black arrow in HN016 showing the vacuolation in the brain. Scale bar: 100 μm (main image) and 12.5 μm (enlarged inset).

To assess the contribution of the T7SS to virulence, tilapia were challenged with HN016, Δ*essC*, or PBS as negative control. Infection with HN016 resulted in rapid mortality, with approximately 90% of tilapia succumbing within 24 h, compared with only 20% in the Δ*essC*-infected group (Figure 5A). By the endpoint of the experiment (14 days post-infection), survival in the Δ*essC*-infected group remained at 60%, whereas only 5% of fish survived in the HN016-challenged group (Figure 5A), confirming severe attenuation of Δ*essC*. To evaluate the effect of the T7SS on the GBS infection burden, the tissue bacterial loads were compared (Figure 5B). Significantly fewer CFU of the Δ*essC* mutant were recovered from the brain, liver, spleen, head kidney, trunk kidney, and hindgut compared with the HN016 strain (Figure 5C–H), indicating markedly impaired systemic dissemination of Δ*essC*.

Histopathological analysis further revealed the extent of tissue damage caused by HN016 or Δ*essC*. In the hindgut, HN016 infection induced severe epithelial denudation and collapse of villous architecture, whereas tissues from the Δ*essC*-infected group displayed normal structure and exhibited increased goblet cells (Figure 5I). Similarly, brain sections from HN016-infected fish showed pronounced pathological alterations, including meningeal inflammation, widespread vacuolation, and a loose, disorganized cerebral parenchyma, while those from Δ*essC*-challenged fish showed normal histological architecture with no observable lesions (Figure 5I). Together, these in vivo findings demonstrate that T7SS is critical for systemic virulence, tissue colonization, and pathogen-induced damage in *S. agalactiae* infection.

### T7SS is required for blood–brain barrier (BBB) penetration

**Figure 6:**
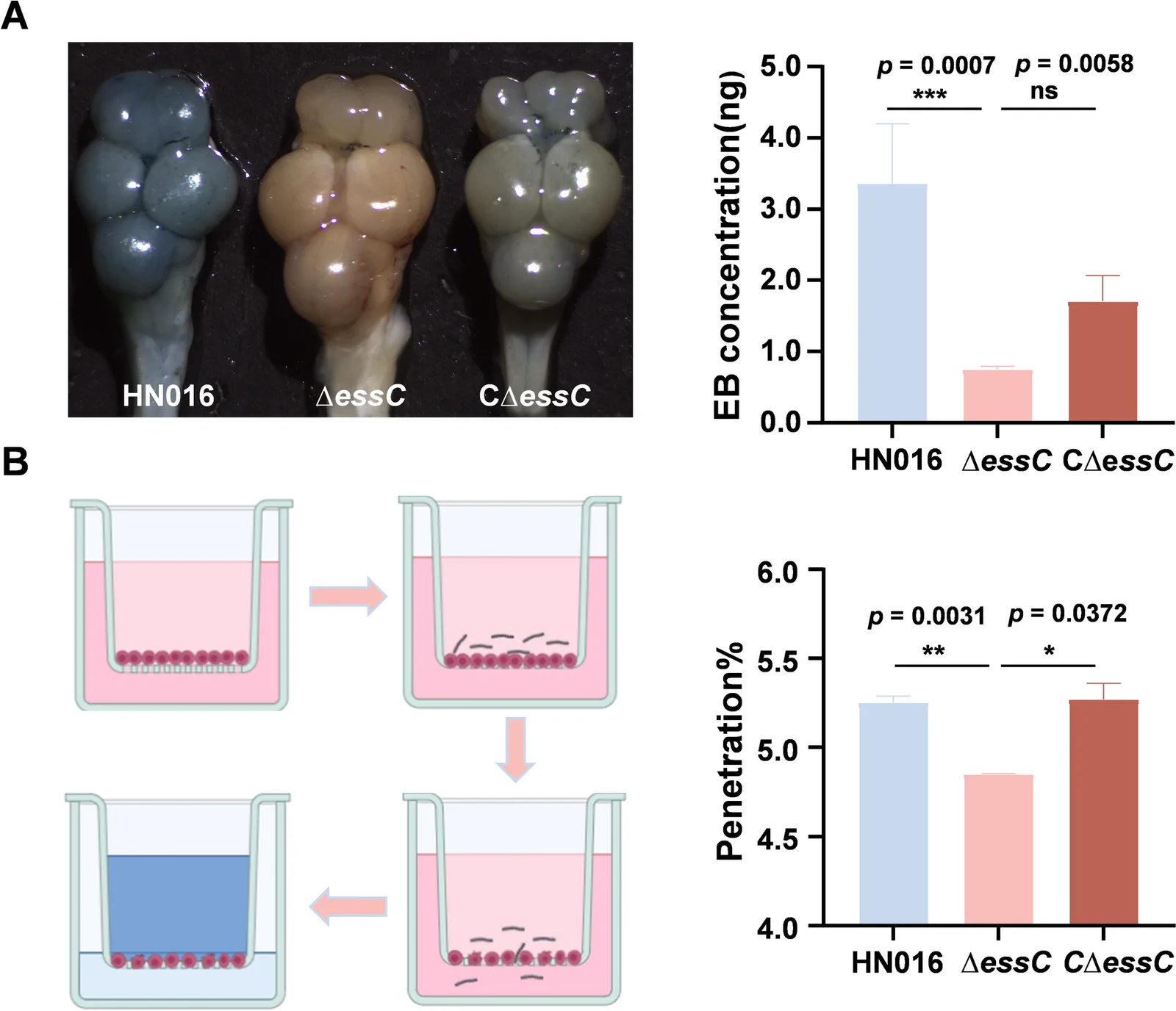
**T7SS is required for blood–brain barrier disruption and penetration.** **A** Evaluation of blood–brain barrier (BBB) integrity using Evans blue (EB) extravasation. Brains were collected at 4 h post‑infection (hpi), and EB was quantified spectrophotometrically. **B** EB penetration across an in vitro human BBB model. hBMEC monolayers grown on Transwell inserts were infected apically with HN016, Δ*essC*, or CΔ*essC* (MOI = 10). The bacteria and cells were co-cultured for 12 h at an MOI of 10, and the percentage of EB that translocated to the basolateral compartment was determined. Data are mean ± standard error of the mean (*n* = 3). *p < 0.05, **p < 0.01, ***p < 0.001; not significant (ns) (one‑way ANOVA with Tukey’s post‑hoc test).

The reduced brain pathology observed in Δ*essC*-challenged fish led us to hypothesize that the mutant might be impaired in crossing the BBB. To test this hypothesis, the EB dye extravasation was used to assess BBB integrity. Infection with HN016 caused pronounced BBB disruption, evidenced by strong dye penetration into brain tissue, whereas infection with the Δ*essC* mutant resulted in significantly less dye permeation over the same time period, an 82% reduction in EB dye level relative to the HN016 (*p* = 0.0007; Figure 6A). Consistent with the in vivo results, infection with the Δ*essC* mutant induced markedly less Evans blue translocation across hBMEC monolayers compared with HN016 (Figure 6B). Collectively, these findings establish an essential role of T7SS in breaching the BBB in vivo and in vitro.

## Discussion

Secretion systems are fundamental to bacterial growth and virulence in bacteria, yet the functional repertoire of the T7SS in the multi-host pathogen *S. agalactiae* remains poorly defined. Recent studies have established T7SS as a versatile machine in Gram-positive pathogens, mediating crucial processes ranging from interbacterial competition and host cell manipulation to the secretion of key virulence factors such as the pore-forming EsxA [2, 31], specialized cell wall hydrolases such as EssH that facilitate effector export across the thick peptidoglycan layer [32], and antibacterial WXG100 toxins [33]. Expanding upon this foundation, we provide the first experimental evidence that T7SS plays a pivotal and previously unrecognized role in Mg^2+^ acquisition and the maintenance of capsular integrity in *S. agalactiae*.

A key discovery of our work is the link between T7SS function and Mg^2+^ homeostasis. Under magnesium-limited conditions, we observed that the Δ*essC* mutant displayed significantly delayed growth, a defect that was fully complemented by WT culture supernatant. EssC is a conserved FtsK/SpoIIIE-type ATPase that mediates substrate recognition and translocation, as demonstrated in mycobacteria, *S. aureus* and *S. agalactiae* [18]. These data suggest that EssC is required for secreting a factor that indirectly facilitates Mg^2+^ acquisition rather than acting as a direct transporter. This finding aligns with a broader paradigm within the T7SS field, where these systems have been increasingly recognized for their roles in nutrient uptake. For instance, the ESX-3 system in *Mycobacterium tuberculosis* is essential for iron and zinc acquisition [34], while the T7SSb in *Staphylococcus aureus* are involved in the import of fatty acids and activation by host-derived fatty acids [35]. Our work therefore extends this emerging theme to magnesium, positioning T7SS as a critical player in ion homeostasis across diverse bacterial pathogens. However, the putative magnesium-associated substrate awaits further characterization.

Furthermore, our transcriptomic and phenotypic analyses reveal a critical mechanistic link between T7SS‑dependent Mg^2+^ homeostasis and capsule biogenesis in *S. agalactiae*. The significant downregulation of key capsule biosynthesis genes (*cpsA*, *cpsB*, and *cpsD*) in the Δ*essC* mutant correlated with reduced capsular protein content, structural instability, and increased cell aggregation (Figures 3 and 4). This connection between a secretion system and capsule biogenesis finds precedent in other pathogens. For instance, in *Mycobacterium* spp., the capsule is enriched with proteins secreted via the T7SSa (ESX) systems. Moreover, T7SSa substrates such as the PE5–PPE4 complex are crucial for iron acquisition, underscoring the role of T7SS substrates in metal homeostasis and structural integrity [11]. By analogy, we propose that T7SS in *S. agalactiae* may secrete specific substrates that facilitate magnesium acquisition. Intracellular Mg^2+^ is known to be critical for the activity of various enzymes, including the capsule synthesis-associated phosphotransferase CpsA [36], Therefore, the increased Mg^2+^ availability resulting from T7SS function would be expected to promote CpsA activity, thereby supporting capsule biogenesis and structural stability. This model is strongly supported by our recent observation that Mg^2+^ depletion, achieved through deletion of the transporter CorA2, leads to a significant reduction in both GBS capsule content and integrity [37]. Together, these data establish a functional link between T7SS-mediated Mg^2+^ homeostasis and capsule biosynthesis.

Bacterial capsule, primarily composed of CPS, is anchored adjacent to the peptidoglycan layer in Gram-positive bacteria and indispensable for invasive pathogenesis. Our study establishes a novel link between T7SS and capsular integrity in *S. agalactiae*. Transcriptomic analysis indicated that the deletion of *essC* disrupts central carbohydrate metabolism, including pathways for amino sugar and nucleotide sugar biosynthesis, which are essential precursors for CPS assembly [38]. Concurrent downregulation of *cpsA*, which encodes an enzyme essential for tethering CPS to peptidoglycan [39], likely further compromises capsular anchoring. This dual impairment in precursor supply and physical tethering provides a mechanistic explanation for the observed "halo" circle in extracellular polysaccharide shedding and the loss of structural coherence in the Δ*essC* mutant (Figure 3D).

In this study, the Δ*essC* mutant exhibited significant reduced adhesion to host cells, lower bacterial burdens in systemic tissues, and ultimately, markedly decreased lethality in vivo. This broad attenuation across virulence phenotypes can be largely attributed to impaired capsular integrity, given that an intact capsule is essential for *S. agalactiae* persistence and invasive disease [40]. Specifically, the diminished serum survival of the Δ*essC* mutant aligns with the established role of the capsule in promoting bloodstream survival across multiple serotypes [41]. Furthermore, recent study in *S. pneumoniae* indicates that the capsule promotes intracellular survival and translocation by enhancing bacterial resistance to oxidative killing [42], a mechanism that may similarly contribute to the attenuated virulence observed in our Δ*essC* mutant. Collectively, this constellation of defects strongly argues that T7SS, through its role in maintaining the capsule, is a global regulator of *S. agalactiae* pathogenicity.

While the specific T7SS effector responsible for maintaining capsular integrity in *S. agalactiae* remains unknown, functional precedent exists in mycobacteria, where the T7SS substrate PPE10 in capsule is essential for capsule stability [12]. This parallel suggests that analogous T7SS effectors in *S. agalactiae* may be identified by comparing the capsular proteomes of the WT and Δ*essC* strains. Indeed, our comparative analysis revealed six distinct protein bands that were markedly reduced in the Δ*essC* mutant (Figure 3G). Mass spectrometry identification of these bands yielded a candidate list of putative T7SS effectors (Additional files 5-9), providing a direct experimental basis for future mechanistic studies.

Our findings establish that T7SS in *S. agalactiae* modulates growth by Mg^2+^ acquisition and virulence by maintenance of capsular integrity. Those results reveal a previously unrecognized vulnerability in streptococcal pathogenesis and suggest that targeting T7SS could represent a novel antivirulence strategy. We acknowledge several limitations of this study. First, while our data strongly support a role for T7SS in magnesium-dependent processes, the precise molecular mechanism by which Mg^2+^ availability remains to be fully elucidated. Second, although capsular impairment appears to be a major contributor to attenuation in the Δ*essC* mutant, we cannot exclude the possibility that T7SS influences additional virulence pathways independent of the capsule. Future studies should be carried out to identify the specific T7SS effector(s) involved in magnesium scavenging and characterizing their interaction with capsule biosynthesis enzymes.

## Conclusions

Our study not only characterizes a novel role for T7SS in *S. agalactiae* pathogenesis but also integrates our findings into a growing body of literature that positions this system as a central hub for coordinating metal ion homeostasis with critical cell envelope processes. By demonstrating a functional linkage between Mg^2+^ acquisition and capsular integrity, we provide a new conceptual framework for understanding T7SS‑mediated virulence. Our findings reveal a previously unrecognized dual role of T7SS in magnesium homeostasis and structural stability, offering novel mechanistic insights into streptococcal pathogenesis and potential target for antivirulence strategies.

## Supplementary Information

**Additional file 1. Primers used in this study.**List of primer sequences used for PCR amplification, mutant construction, and qRT-PCR analysis

**Additional file 2. Construction and validation of the ΔessCmutant inStreptococcus agalactiaeHN016.**Thestructure of EssC ofMycobacterium tuberculosis(A) or S. agalactiae (B) predicted by Swiss-Model software. (C) Schematic representation of the T7SS genomic locus in HN016 (accession NZ_CP011325). Genes encoding the EsxA1 or EsxA2 proteins are shown in purple; the core ATPaseessCis highlighted in red. (D) PCR verification of theessCdeletion using internal/external primers. Lanes 1 show PCR products form ΔessCclone with external primers, lane 2 show PCR products form ΔessCclone with internal primers. (E) PCR verification of the complemented strain CΔessCusing M13F/M13R primers. Lane 3 show PCR products from a CΔessCclone with M13F/M13R primers. N: negative control without template. P: positive control with a WT HN016 genomic DNA as template. M: DNA marker

**Additional file 3. Growth curves of HN016, ΔessCmutant and complemented strain CΔessCwere monitored in THB.**Bacterial growth was monitored by measuring optical density at 600 nm (OD) overtime. Data represent the mean ± standard deviation (SD) from three independent biological replicates

**Additional file 4. qRT‑PCR validation of selected down-regulated expression genes of DEGs (spx,03175,gapandraiA) in HN016 and the ΔessCmutant.**Expression levels are normalized to the housekeeping genegyrA. Data are mean ± SEM of three biological replicates (*p< 0.05, **p< 0.01, ***p< 0.001, Student’st‑test)

**Additional file 5. Mass spectrometry analysis of band-1 in Figure 3G.**The protein band indicated by the arrow 1 in Figure 3G was excised from an SDS-PAGE gel, digested with trypsin, and analyzed by LC-MS/MS using a Thermo LTQ FT. Protein identification was performed against theS. agalactiaeHN016 database. Only proteins with at least two unique peptides per identification, a false discovery rate (FDR) < 1% at both the peptide and protein levels, and a peptide score ≥ 95% confidence interval were considered for positive identification. The top candidate hits are listed with their corresponding gene names, accession numbers, molecular weights (kDa), sequence coverage (%), and the number of unique matched peptides

**Additional file 6. Mass spectrometry analysis of band-2 in Figure 3G.**The protein band indicated by the arrow 2 in Figure 3G was excised from an SDS-PAGE gel, digested with trypsin, and analyzed by LC-MS/MS using a Thermo LTQ FT. Protein identification was performed against theS. agalactiaeHN016 database. Only proteins with at least two unique peptides per identification, a false discovery rate (FDR) < 1% at both the peptide and protein levels, and a peptide score ≥ 95% confidence interval were considered for positive identification. The top candidate hits are listed with their corresponding gene names, accession numbers, molecular weights (kDa), sequence coverage (%), and the number of unique matched peptides

**Additional file 7. Mass spectrometry analysis of band-3 in Figure 3G.** The protein band indicated by the arrow 3 in Figure 3G was excised from an SDS-PAGE gel, digested with trypsin, and analyzed by LC-MS/MS using a Thermo LTQ FT. Protein identification was performed against theS. agalactiaeHN016 database. Only proteins with at least two unique peptides per identification, a false discovery rate (FDR) < 1% at both the peptide and protein levels, and a peptide score ≥ 95% confidence interval were considered for positive identification. The top candidate hits are listed with their corresponding gene names, accession numbers, molecular weights (kDa), sequence coverage (%), and the number of unique matched peptides

**Additional file 8. Mass spectrometry analysis of band-4 in Figure 3G.** The protein band indicated by the arrow 4 in Figure 3G was excised from an SDS-PAGE gel, digested with trypsin, and analyzed by LC-MS/MS using a Thermo LTQ FT. Protein identification was performed against theS. agalactiaeHN016 database. Only proteins with at least two unique peptides per identification, a false discovery rate (FDR) < 1% at both the peptide and protein levels, and a peptide score ≥ 95% confidence interval were considered for positive identification. The top candidate hits are listed with their corresponding gene names, accession numbers, molecular weights (kDa), sequence coverage (%), and the number of unique matched peptides

**Additional file 9. Mass spectrometry analysis of band-5 in Figure 3G.** The protein band indicated by the arrow 5 in Figure 3G was excised from an SDS-PAGE gel, digested with trypsin, and analyzed by LC-MS/MS using a Thermo LTQ FT. Protein identification was performed against theS. agalactiaeHN016 database. Only proteins with at least two unique peptides per identification, a false discovery rate (FDR) < 1% at both the peptide and protein levels, and a peptide score ≥ 95% confidence interval were considered for positive identification. The top candidate hits are listed with their corresponding gene names, accession numbers, molecular weights (kDa), sequence coverage (%), and the number of unique matched peptides

## Data Availability

Data will be made available on request. The data that support the findings of this study are openly available via Figshare at 10.6084/m9.figshare.31294984.
