# Supplementary material for: Type VII secretion system promotes Streptococcus agalactiae virulence through magnesium acquisition and capsule maintenance
Source: Vet Res. 2026 Aug 27;57:154. doi: 10.1186/s13567-026-01816-9 (PMC13520451; doi:10.1186/s13567-026-01816-9)
Supplement: Supplementary file 1 [file 13567_2026_1816_MOESM1_ESM.docx]

**Additional file 1.** Primers used in this study.

| **Primer** | **Sequence (5' to 3')** | **Use** |
| --- | --- | --- |
| pSET4s-*essC* up-F | cggccagtgaattcgagctcCACAGAAGTACGAGTTGGCC (Sac l) | Upstream fragment of *essC* |
| pSET4s-*essC* up-R | GTATTTTTGCCATTCTCTCTCCTCATCCACCTACCTACTTTTAGTTTTAGAAG | Upstream fragment of *essC* |
| pSET4s-*essC* down-F | CTTCTAAAACTAAAAGTAGGTAGGTGGATGAGGAGAGAGAATGGCAAAAATAC | Downstream fragment of *essC* |
| pSET4s-*essC* down-R | gcatgcctgcaggtcgacGTCGCTCTTGGACAAGTCCT (Sal l) | Downstream fragment of *essC* |
| ∆*essC*-in-F | TCCACTTGGCTTACGTGGTC | Fragment within the in-frame deletion *essC* |
| ∆*essC*-in-R | GTGTGGCAGGTCCTTGAAGA | Fragment within the in-frame deletion *essC* |
| ∆*essC*-out-F | TGTTGGCAGCGGATACTTCA | Fragment covering the in-frame deletion *essC* |
| ∆*essC*-out-R | TCCATCAAAGGCATGCGACA | Fragment covering the in-frame deletion *essC* |
| pSET2-*essC*-F | gattacgccaagcttgcatgcctgcagGGTGGATATGTTAGTTATTTGTTATTATCAATC (Pst I） | Complementary plasmid construction |
| pSET2-*essC*-R | gtaaaacgacggccagtgaattcCTCTCCTCTCTAGTCTATGCTG (EcoR I) | Complementary plasmid construction |
| M13F | CGCCAGGGTTTTCCCAGTCACGAC | For insert screening and plasmid presence |
| M13R | CAGGAAACAGCTATGACC | For insert screening and plasmid presence |
| pSET2-uridine phosphorylase-EGFP-F | CgccaagcttgcatgcGATGCAGAATTATTCAGGTGAAGTC (sph I) | Fragment covering the uridine phosphorylase |
| pSET2-uridine phosphorylase-EGFP-R | CCCTCCGCCACCTGACCCTCCGCCACCTTGTGATTTATCATTTTCAATAAGTGTGCG | Fragment covering the uridine phosphorylase |
| EGFP-F | GTGGCGGAGGGTCAGGTGGCGGAGGGTCCGGTGGCGGAGGGTCG atggtgagcaagggcg | Fragment covering the EGFP |
| EGFP-R | cgacggccagtgaattcttacttgtacagctcgtccatgc (EcoR I) | Fragment covering the EGFP |
| Qpcr *cpsA*-F | TCAACTGGACAACGCTTCAC | For qRT-PCR |
| Qpcr *cpsA*-R | AAGTTGAGCTCCTGGCATTG |  |
| Qpcr *cpsB*-F | CGGGCTCTTTTCTTAAAGCGT |  |
| Qpcr *cpsB*-R | TACACCAGTCGTTGCGCATA |  |
| Qpcr *cpsD*-F | TCAATGCGATCCGTACAAAC |  |
| Qpcr *cpsD*-R | GTGGATTTTCCTTCCCCTTC |  |
| Qpcr *cpsE*-F | TGCTCATATGTGGCATTGTG |  |
| Qpcr *cpsE*-R | AGAAAAGATAGCCGGTCCAC |  |
| Qpcr *spx*-F | AGCGAGGTAGAAAAGCACGA |  |
| Qpcr *spx*-R | TCGTTATGCTAAGGCGCTCA |  |
| Qpcr *gap*-F | GGAACAGGAACACGTTGTGC |  |
| Qpcr *gap*-R | TCCACGCATACACTGGTGAC |  |
| Qpcr *HPr*-F | AGGTATTCACGCACGTCCAG |  |
| Qpcr *HPr*-R | AGCACCTTGACCAACACCAA |  |
| Qpcr-g*rpE*-F | CGAGCGTGCTGATGAGTTTG |  |
| Qpcr g*rpE* -R | GCGTTGAAGTTGCTGACGTT |  |
| Qpcr-*raiA*-F | TGAGCCAGATGAAGAAGCAGT |  |
| Qpcr *raiA* -R | GTTCGCCATCTTCACGCTTG |  |
| Qpcr-*03175*-F | GTTGAAACGGTCCACACGTC |  |
| Qpcr *003175*-R | TCGAAGGTGAAACTTACCCACT |  |
| Qpcr gyrA-F | AGGTTTACTTGTGGCGCTTG |  |
| Qpcr gyrA -R | TCTGCTTGAGCAATGGTGTC |  |
